# Syphilis as a Cause of Abortion*Translated from *La Clinique*, Decembre, 1896, by L. B. Grandy, M.D., Atlanta.

**Published:** 1897-01

**Authors:** J. A. Ouimet

**Affiliations:** Montreal, Can.


### SYPHILIS AS A CAUSE OF ABORTION.*

By DR. J. A. OUIMET,

Montreal, Can.

Syphilis is one of the causes of abortion. It is to-day universally admitted that among syphilitic women, labor at term is the exception when the malady is of recent date, and has not been combated by treatment vigorously applied and sufficiently prolonged.

When I was spending a year in the service of Professor Tarnier, of Paris, I was struck with some of the accidents caused by syphilis, and I then undertook some researches, the results of which I am now going to publish. Syphilis being a cause of considerable mortality among infants, indeed, according to Professor Fournier, one of the greatest factors of mortality in children, if in showing the remedies for the disease we are able to point out also the necessity of treatment during pregnancy, we believe that we will contribute our feeble part toward saving the lives of some children very often condemned to death before they are born. Numerous statistics have been prepared as to the frequency of abortions caused by syphilis, and all show with terrible eloquence that this disease is a potent cause of abortion and premature labor. It plays an important rôle in the mortality of infants, especially in cities. If in the country, it is a disease not yet very widespread, I am able to state that it is, unfortunately, too frequent in small cities, and very prevalent in large cities. Professor Pinard reports that out of 687 women delivered at the obstetrical clinic in 1882, thirty-four were affected with syphilis—a little less than five per cent. If one examines the influence of syphilis on pregnancy, this influence will be found to vary with the conditions under which the disease presents itself in a particular case. The absolute frequency of abortion or of premature labor is difficult to fix in an exact manner. The proportion of accouchements before term among syphilitic women is, according to Diday, 61 per cent.; Stoltz, 66 per cent.; Hecker, 27 per cent.; Fournier, 47 per cent.;

*Translated from *La Clinique*, Decembre, 1896, by L. B. Grandy, M.D., Atlanta.

Pilem, 36 per cent. Out of a large number of statistics furnished by different authors, one may accept 36 per cent. as an approximate estimate. To be very exact, it is necessary to take into account the age of the syphilis, its intensity, and the nature and effects of the treatment that has been pursued. This proportion of 36 per cent. is too small, if one applies it to those women who, at the time of conception, were in the full evolution of syphilis, or to those who during the first months of their pregnancy have exhibited intense manifestations of the disease. On the other hand this proportion is too great if it applies to those women in whom, at the time of conception, the syphilis is already old, especially if the patient has been well cared for; for the accidents of abortions caused by syphilis are obedient to the law of decadence, the law of Diday, according to which the intensity diminishes as the disease grows older. At first there are successive abortions, followed by premature births, and finally by labors at term. In the last two cases the age of the syphilis has a great influence upon the life of the child, and here we find the same decadence: at first dead infants, next living children, but tainted with congenital syphilis, and finally healthy children.

At what period of pregnancy is abortion among syphilitics most frequent? While it is more or less frequent at all periods, nevertheless it becomes more so as one approaches the seventh month, the maximum of frequency being between the sixth and seventh month; one finds it less often between the seventh and ninth month. Bertin, Diday and Deville are of the opinion that the infection of the fetus is a frequent cause of abortion at the fifth, sixth and seventh month; in the second half of pregnancy, according to Olshausen. The period of pregnancy at which expulsion of the product of conception occurs certainly varies according to the age and gravity of the syphilis; it also depends upon whether or not the woman has submitted to methodical treatment, as we shall see when we discuss the results obtained by treatment. According to Diday, the abortion corresponds to the maximum of the potency of the syphilitic virus. It is especially to be feared in the early period of syphilis, and Diday even declares that all women not treated abort regularly during the first three years of their disease. It is not rare to see in a syphilitic woman the first pregnancy terminated by abortion; subsequent pregnancies may go to term. The latter modification, frequent among women who have submitted to proper treatment, is also observed among those who have not been treated, thus confirming the general law proposed by Diday. Finally one must remember that there are degrees of syphilis. There is benign or light syphilis, and the intense or malignant.

Among women who have never submitted to specific treatment, it is common then to have repeated abortions. There is a series of abortions recurring in the same women, to which has been given the name *avortements à repetition.* In the etiology of these repeated abortions, the cause of which one may not be able to determine, syphilis, without doubt, occupies an important place. Trousseau said: "When you are called to a woman with whom abortion or premature labor is a habit, you will be wrong if you do not consider syphilitic infection among the possible causes when you are making up your judgment." The repetition of abortions without manifest cause may be a sign proclaiming the presence of syphilis, and Depaul and Fournier, in the presence of such abortions, believe themselves authorized in employing specific treatment, the results of which are often conclusive. These abortions may be repeated a considerable number of times, six not being exceptional. Fournier has reported the case of one woman who aborted nine times, and another who had ten successive abortions or premature labors, and who was delivered at term in her eleventh labor of a syphilitic child, which died after four days.

Whether the abortion depends upon utero-placental lesions, or is caused by the fetus itself, the mechanism is always the same. The woman perceives that the movements of the fetus diminish from day to day, soon they cease altogether, and the sympathetic troubles of pregnancy which had existed up to that time disappear also. The belly goes down, the breasts diminish in size, there is a feeling of heaviness in the loins and in the lower abdomen, and also a sensation of an inert body in the uterus which changes its position with the movements of the woman, obeying the laws of gravity. Soon uterine pains set up and everything passes off as an ordinary abortion.

*The Causes of Abortion in Syphilitics.* Almost all observers attribute the cause of abortion to the fetus itself. Syphilis certainly places the fetus in a condition of lowered vitality, and while it may not be necessary for the fetus to be dead in order to be expelled, nevertheless it is a common observation in the great majority of abortions among syphilitics that the fetus is dead, and more often macerated. According to Blaise, the death of the fetus may be caused by a direct and primitive poisoning by a morbid principle. It is analogous to that which occurs in lead poisoning. Syphilis, like lead-poisoning, may cause abortion by inflicting injury upon the health of the mother, and by exercising a direct action upon the product of conception. The analogy of syphilitic infection is even greater with certain virulent affections, such as measles, scarlatina, and small-pox, which can be transmitted from mother to fetus. Often the fetus is dead, almost always it is affected with organic lesions, more or less grave, which certainly are concerned in the premature expulsion. The abortion, perhaps, shows syphilitic involvement of the wall of the uterus, of the placenta, or of its membranes. There is a syphilitic placenta which presents special features, and this placenta is seen only in cases of congenital fetal syphilis. These lesions may be on either the fetal or maternal surface of the placenta. Syphilis of the maternal surface of the placenta may cause death of the fetus by producing a thickening, more or less extended, of this part of the placenta; this produces first a compression and then an atrophy of the villi.

*The influence of paternal syphilis on pregnancy and the product of conception.* Has the father a direct influence on the product of conception—that is, can a father transmit syphilis to a fetus without infecting the mother? This question has been the subject of many a controversy. While the older syphilographers attributed to paternal influence a preponderating and often an exclusive part in the hereditary transmission of syphilis, other authors more modern, but equally exclusive, deny to the father any influence whatever. To-day the opinion which generally prevails is a sort of middle-ground; in other words, that the influence of the father is far from being as common as that of the mother. Fränkel admits that the syphilitic virus of the father may act directly upon the ovum through the semen, and that in this case the mother may remain healthy. Syphilis in the father certainly has an effect upon pregnancy, for there are numbers of cases in which the father has been syphilitic at time of marriage and the mother has had first an ovular abortion followed later by an embryonic. The father then takes mercurial treatment, and the mother has a premature labor; he continues treatment, and she goes to term. Other cases no less demonstrative are those in which the mother, having had children one after another and at term by a first husband, has successive abortions in a second marriage, and in which specific treatment of the father has resulted in the birth of a healthy child at term. Fournier says that the fetus of a syphilitic father and a healthy mother is liable by the fact of the paternal syphilis to die before term. Out of 43 unions, in which the father alone was syphilitic, and which furnished 105 infants, Kassowitz found 25 terminations of pregnancy before term, and 80 births at term with syphilis. Fournier formulates the conclusion that "given a syphilitic father and a healthy mother there are some chances for the infant issue of this couple to be born exempt from syphilis." There are many more chances if the father submits to mercurial treatment before the conception. If the age of the syphilis in the mother plays an important part in terminating the pregnancy, this is particularly the case with the father. Some authors are of the opinion that tertiary paternal syphilis does not have any influence upon the product of conception; that time diminishes the potency of the syphilitic diathesis.

The mother may become syphilitic at the time, or soon after she becomes pregnant. In this case one can easily see what will happen to the product of the conception; she will either abort of a dead or macerated fetus, or she will be delivered of an infant which will have the finest chances to inherit syphilis from its mother. When the father and mother are both syphilitic, of course the conditions for heredity are doubly favorable; to a syphilitic ovule is added the action of syphilitic spermatozoids, the development taking place in a medium of the same nature; a biparental heredity.

*The treatment of syphilis during pregnancy.* In the presence of the marvelous results obtained by mercury in the treatment of syphilitic pregnant women, one is astonished at the tardy acceptance of this idea and at the resistance which it has encountered. Mauriceau, a famous observer, recognized the excellent effects of mercury on a syphilitic pregnant woman, and prescribed it. Bertin said that a pregnant woman with syphilis could perhaps be radically cured during her pregnancy, and the infant perhaps be saved from the disease by the mercurial treatment; and he added, an anti-venereal treatment, prudently administered, does not produce abortion, as the older doctors feared. Although in some cases abortion has happened in the course of a mercurial treatment, it is more often the effect of the disease than of the mercury. Observation has proven that infected pregnant women have aborted more often when they have submitted to no treatment than when they were treated during pregnancy. To-day the majority of doctors employ mercury without inconvenience in the treatment of the syphilitic pregnant woman. Far from thinking that it may produce an abortion, one now knows that very often it is the only means of preventing it. Benjamin Bell said that mercury, properly administered, almost always succeeded in preventing the abortion. He also said that specific treatment of the pregnant woman could produce two results: (1) diminish the frequency of the abortions, and (2) often preserve the infant from syphilitic infection.

The pregnant woman tainted with syphilis demands all the solicitude of the physician; she deserves to be treated with care, attention and vigilance. Many times, says Fournier, have I obtained from specific medication the first and inestimable success in preventing abortion, in carrying the woman to term. The infant born in these conditions, it is true, does not escape syphilis, but he is born viable and susceptible of living with the disease, and of being cured by subsequent treatment. Indeed, one has seen in such circumstances infants born healthy, free from every syphilitic symptom.

One ought to commence the treatment as soon as the syphilis is diagnosed, and indeed, if one has the good fortune to be called to diagnose a case of syphilis in parents, before conception occurs; one will then have more chances of preserving the life of the child. Fournier is of the opinion that as soon as one has recognized the presence of syphilis in a pregnant woman, he should proceed without hesitation; introduce treatment immediately. The later treatment is introduced in the course of pregnancy, the less will be the chances of saving the life of the infant, and still less of assuring its health. Diday says that when one has recognized syphilis in a pregnant woman, there is only one thing to do—prescribe mercury immediately, and push its administration with all the vigor which comports with the constitution of the patient and the manner in which she responds to treatment. How long should the treatment be kept up? At least as long as pregnancy lasts, as long as the disease may cause abortion. On this point there are some rules laid down by that master, Professor Fournier: "One is able to cure syphilis, or rather, to impose silence on the manifestations of the disease, only at the price of treatment long, very long, extending over many years. This treatment, to be efficacious, needs to be methodical; it should be alternately interrupted and resumed, and by different methods. In a word, syphilis is a chronic malady which is eliminated only by chronic treatment."

The treatment of syphilis during pregnancy is the same as that without pregnancy, unless there is some disturbance of the digestive organs which will require a certain prudence. It is certain that one should combat all syphilitic manifestations in full measure, but the mercurial treatment—the only efficacious treatment—being able to cause vomiting and diarrhea in some women, should be handled with precaution in order not to irritate the stomach and intestines, and thus perhaps cause abortion. One should be able to bring to bear on the pregnant woman all the modes of mercurial treatment as well as the various mercurial preparations. Mercury alone, or when it is necessary, combined with iodide of potassium, may be administered by the digestive organs, the stomach or rectum, by the respiratory organs, by the skin or by the subcutaneous method. The most common methods of treatment are by the mouth and mercurial frictions. One preparation very commonly employed, is the solution of Van Swieten,* although with a pregnant woman

*Liq. hydrargyri oxymuriatis: Hydrarg. bichloridi, ammon. chlorid. aa. gr. x, aquæ oi.

the corrosive sublimate which this solution contains may irritate the gastro-intestinal mucous membrane and soon may not be tolerated. Fournier gives preference to the protoiodide of mercury, which is pleasant and better borne by most patients. He prescribes five to ten centigrams (gr. $\frac{3}{4}$–$1\frac{1}{2}$) per day, taken at meal times. The phenate of mercury often gives good results. One may employ also the syrup of Gibert, composed of the biniodide of mercury and the iodide of potash. If the ingestion of mercury cannot be long continued with some women, it will be well to resort to mercurial inunctions along with the internal administration. It is always useful and often necessary to add some tonic: wine, wine of quinine, elixir of Garus, iron, arsenic, etc., in order to maintain the tone of the digestive apparatus.

*Conclusions.* Syphilis is a potent cause of abortion. The abortion is caused by lesions of the fetus itself, or of its adnexæ.

It occurs ordinarily about the seventh month. The father alone being syphilitic, he may transmit the disease to the product of conception; he is much more liable to do so if the syphilis is recent at the time of conception.

The mother may bring into the world a syphilitic infant, and remain herself immune.

When the father and mother are both syphilitic, the infant rarely escapes infection.

A syphilitic pregnant woman has a greater chance of bringing into the world a healthy infant when the syphilis is old. The nearer the primary infection occurs toward the end of pregnancy the better the infant's chances of escaping infection.

The infant born of a syphilitic mother may present manifest syphilitic lesions, or it may be healthy in appearance, and become syphilitic after some months, or even years.

Mercurial treatment instituted at the beginning of pregnancy may enable the mother (1) to carry the pregnancy to term, or (2) to bring into the world a living infant, though perhaps syphilitic—or (3), in some cases to bring into the world a living infant without lesions. Sometimes an infant born of syphilitic parents remains free from the disease, when the mother has been treated during pregnancy.

When the father is syphilitic, and the mother becoming pregnant, submits to mercurial treatment, there are good chances for the pregnancy to end at term in the birth of a healthy infant.

